# Pericardial Tamponade and Berger’s Disease: An Unusual Association

**DOI:** 10.7759/cureus.41281

**Published:** 2023-07-02

**Authors:** Siva Naga S Yarrarapu, Parth Shah, FNU Arty, Jayasree Ravilla, Medha Ghose, Mahrukh A Khan, David Anwar

**Affiliations:** 1 Internal Medicine, Monmouth Medical Center/Rutgers University, Long Branch, USA; 2 Hospital Medicine, Tower Health Medical Group, Reading, USA; 3 Cardiology, Monmouth Medical Center/Rutgers University, Long Branch, USA

**Keywords:** berger’s disease, tamponade, pericardial effusion, cardiac tamponade, iga nephropathy

## Abstract

Cardiac tamponade is considered a medical emergency because a patient can deteriorate easily and die of cardiac arrest if the fluid is not drained immediately. The most common etiologies are the same as pericarditis as fluid accumulates due to pericardial inflammation, including infection, malignancy, trauma, iatrogenic, autoimmune, post-myocardial infarction, radiation, and renal failure. Although the treatment is pericardiocentesis or pericardial window, finding the etiology responsible for the development of pericardial effusion is important. Here, we describe the case of a 40-year-old female who presented to the emergency department with a chief complaint of severe epigastric pain of a two-day duration that was associated with multiple episodes of nausea, vomiting, dysphagia, and severe shortness of breath (New York Heart Association III). The patient was eventually diagnosed with cardiac tamponade as a cause of her dyspnea, as a two-dimensional cardiac echocardiogram detected a large pericardial effusion (>2 cm) with echocardiographic indications for cardiac tamponade with severe pulmonary hypertension. The patient underwent a therapeutic pericardial window with drainage of 250 mL of pericardial fluid. Ultrasound of the abdomen focusing on the kidneys showed an atrophic and echogenic right kidney with a bidirectional flow in the hepatic veins, suggestive of right heart failure. Subsequently, she underwent a kidney biopsy that showed diffuse mesangial proliferative glomerulonephritis with segmental sclerosing features consistent with IgA nephropathy, associated with tubular atrophy, interstitial fibrosis, interstitial inflammation, and moderate arteriosclerosis. The patient was diagnosed with stage V chronic kidney disease secondary to IgA nephropathy. IgA nephropathy is usually common in Caucasian or Asian males in their teens and late 30s, with hematuria as a usual presentation. This case is unique as cardiac tamponade with renal failure is rarely the presenting symptom of IgA nephropathy.

## Introduction

Pericardial effusion is an accumulation of fluid in the pericardial space, which can be acute if it develops rapidly or subacute and chronic if it develops gradually. It can be asymptomatic or can present in the form of cardiac tamponade depending on the amount of fluid accumulation and its effect on cardiac function. Cardiac tamponade develops when fluid in the pericardial cavity is severe enough to decrease cardiac output. It is considered a medical emergency because a patient can deteriorate easily and die of cardiac arrest if the fluid is not drained immediately [1,2]. The most common etiologies are the same as pericarditis as fluid accumulates due to pericardial inflammation, including infection, malignancy, trauma, iatrogenic, autoimmune, post-myocardial infarction, radiation, and renal failure [3]. The usual causes of cardiac tamponade in adult patients are viral or autoimmune. The classic Beck’s triad is only present in 10-40% of cases [4]. Classic EKG findings are low-voltage QRS complexes and electrical alternans due to the swinging of the heart in a pericardial effusion [5]. Echocardiogram is the best imaging test to diagnose cardiac tamponade. Although the treatment is pericardiocentesis or pericardial window, finding the etiology of pericardial effusion is important [1].

## Case presentation

**Figure 1:**
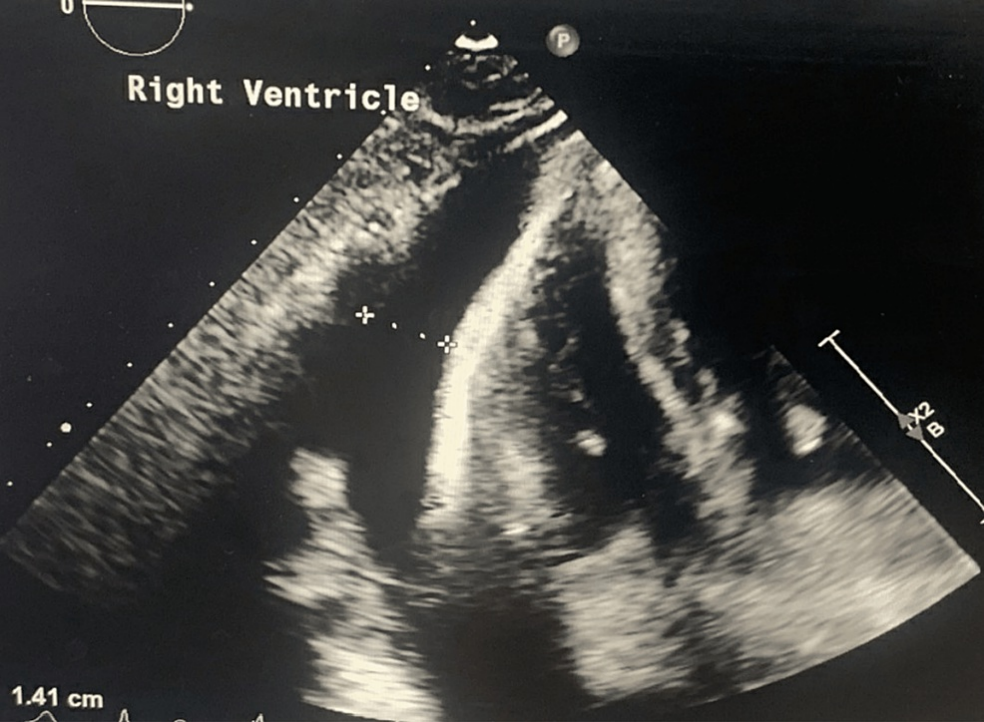
Image showing pericardial effusion (>2 cm).

**Figure 2:**
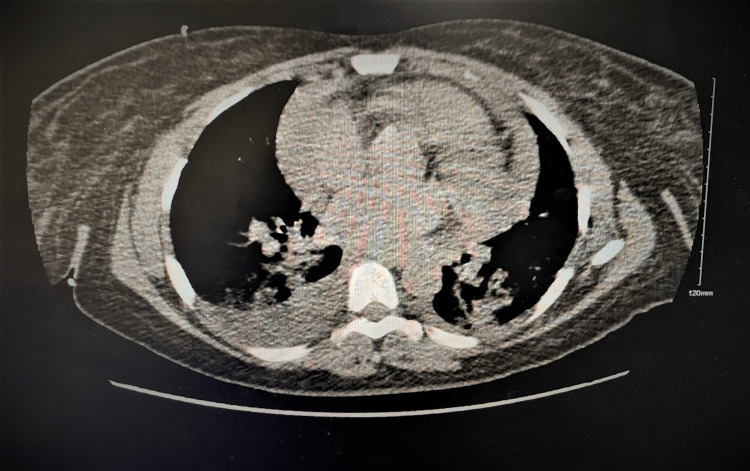
Concomitant moderate right-sided and trace left-sided pleural effusions.

A 40-year-old female presented to the emergency department (ED) with the chief complaint of severe epigastric pain of a two-day duration that was associated with multiple episodes of nausea, vomiting, dysphagia, and severe shortness of breath (New York Heart Association III). She endorsed having mild chest pain for the past two years with elevated blood pressure, for which she never sought treatment. On admission, blood pressure was 216/119 mmHg, refractory to anti-hypertensive medications, white blood cell was 13.2 × 10^9^/L, BUN was 137 mg/dL, and creatinine was 18.7 mg/dL. She underwent an ultrasound of the abdomen that showed pericardial effusion. On subsequent two-dimensional cardiac echocardiogram, a large pericardial effusion (>2 cm) was noted with echocardiographic indications for cardiac tamponade with severe pulmonary hypertension (Figure 1). CT of the chest showed concomitant moderate right-sided and trace left-sided pleural effusions (Figure 2).

The patient underwent a therapeutic pericardial window the next day with the placement of three pericardial and one right-sided pleural chest tube. About 250 mL of pericardial fluid and 120 mL of pleural fluid were drained. At the same time, the patient underwent a repeat ultrasound of the abdomen focusing on the kidneys showing an atrophic and echogenic right kidney with a bidirectional flow in the hepatic veins, suggestive of right heart failure. Subsequently, she underwent a kidney biopsy that showed diffuse mesangial proliferative glomerulonephritis with segmental sclerosing features consistent with IgA nephropathy, associated with tubular atrophy, interstitial fibrosis, interstitial inflammation, and moderate arteriosclerosis. The patient was started on scheduled hemodialysis, resulting in the creatinine settling to around 6.35-6.86. The patient was diagnosed with stage V chronic kidney disease (CKD) secondary to IgA nephropathy.

## Discussion

IgA nephropathy is usually common in Caucasian or Asian males in their teens and late 30s, with hematuria as a usual presentation. This case is unique as cardiac tamponade with renal failure is rarely the presenting symptom of IgA nephropathy.

Pericarditis in patients with end-stage renal disease can be divided into two types, namely, uremic pericarditis and dialysis pericarditis. Uremic pericarditis is defined as the occurrence of clinical symptoms before or within eight weeks of the start of renal replacement therapy (RRT). Dialysis pericarditis is defined as the development of clinical symptoms after more than eight weeks of being stabilized on RRT [6].

The development of uremic pericarditis has been associated with the buildup of toxic metabolic end-products, fluid, and electrolyte imbalance [7]. Interleukin‐1-mediated injury consequent to the toxin buildup was hypothesized to cause pericardial injury [8]. High albuminuria in CKD is linked to inflammation, fibrinolysis, and dyslipidemia. Excess urine albumin excretion may contribute to pericarditis development by increasing endothelial permeability [9,10]. Insufficient dialysis, whether in stable people or those with heightened catabolic activity due to underlying comorbidities, is likely to cause dialysis pericarditis. This condition has become more prevalent among patients experiencing vascular access issues, resulting in missing or inadequate treatments [11].

A patient with pericarditis can present with anterior chest pain that intensifies during inspiration and is often accompanied by the presence of pericardial friction or rub. A range of diagnostic and therapeutic modalities are available to diagnose and treat uremic pericarditis. Patients with uremic pericarditis might not exhibit the characteristic EKG findings of diffuse ST elevation due to a lack of inflammatory cell infiltration into the myocardium [12]. A chest X-ray can assist in determining the size of the heart and the presence of pericardial effusion [13]. There is a substantial likelihood that the enlarged heart silhouette is not due to fluid accumulation even when cardiomegaly is seen. In the absence of lung alterations, a quick change in the size of the heart compared to prior imaging denotes pericardial effusion [14]. Other imaging modalities, such as cardiac MRI and CT, can be used to identify complex or suspected pericarditis. Contrast is, however, avoided in patients with advanced renal disease [15]. In both uremic and dialysis pericarditis, pericardial fluid is constituted by an exudate consisting of mononuclear cells [16].

A high prevalence of asymptomatic pericardial effusion was noted in more than 70% of the patients with uremic and dialysis pericarditis [17]. In a study involving 150 hemodialysis patients, 62% were found to have pericardial effusion, while about 7% exhibited signs of pericarditis or EKG changes [18]. Cardiac tamponade was found to occur in approximately 20% of dialysis pericarditis patients [19]. Dialysis-associated hypotension is observed in 60% of patients with tamponade or pre-tamponade, in contrast to 6% of those who do not present with these conditions [20].

The primary and crucial initial management for patients not on dialysis is to initiate dialysis as early as possible and for those already on dialysis to augment dialysis (class IIa) [21]. Resolution of pericarditis usually occurs within one to two weeks of adhering to frequent dialysis. Assessing the success of dialysis using clinical or laboratory criteria can be challenging; hence, intensive dialysis should continue until the pericardial friction rub disappears. A poor response to intensive dialysis can manifest in around 25% of the cases, prompting early consideration of alternative medical therapies such as nonsteroidal anti-inflammatory drugs or corticosteroids (class IIb) [22]. One must be careful with colchicine due to poor clearance with dialysis (class IIIc) [21]. Low-dose corticosteroids (prednisone 0.2-0.5 mg/kg/day) may be considered when the above-mentioned medical therapies fail [23].

According to the 2015 European Society of Cardiology guidelines, class IC recommendations include pericardiocentesis and pericardial window for recurrent cases, without sufficient data to determine which is better [21]. A pericardial window can be a suitable option, as it allows for obtaining a pericardial biopsy to rule out other disease processes. Additionally, a pericardial window provides the advantage of immediate relief from tamponade when pericardiocentesis is ineffective. However, the recurrence of pericardial effusion secondary to the uremic state can only be addressed through concomitant dialysis. For patients with a large pericardial effusion, cardiac tamponade, or pre-tamponade physiology, a pericardial window, or alternatively pericardiocentesis, can serve as a temporary measure before dialysis can be performed [24]. For constrictive pericarditis, a pericardiectomy is the definitive management, with a success rate exceeding 97%, and less than 1% recurrence [25].

## Conclusions

Cardiac tamponade with renal failure is a rare presentation of IgA nephropathy. IgA nephropathy is usually common in Caucasian or Asian males in their teens and late 30s, and thus, physicians should have a low threshold for suspicion of uremic causes for inflammation of or effusion in the pericardial sac.
